# Hyperthermic intrathoracic chemotherapy in patients with malignant pleural mesothelioma after cytoreductive surgical procedures: a systematic review

**DOI:** 10.1186/s12957-025-03748-8

**Published:** 2025-04-09

**Authors:** Hany Hasan Elsayed, Mohamed Elanany Elsaid Elanany, Mohamed Tarek ElSayegh, Aly Sherif Hassaballa, Mohammed Abdel-gayed

**Affiliations:** 1 Thoracic Surgery Department, Ain Shams University Hospital, Cairo, Egypt; 2 Cardiothoracic Surgery Department, Ain Shams University Hospital, Cairo, Egypt; 3 Military Hospital, Southern Region, Kingdom of Saudi Arabia

**Keywords:** Mesothelioma, HITHOC, Intraoperative chemotherapy, Hyperthermic intrathoracic chemotherapy, Cytoreductive surgery

## Abstract

Surgery-based multimodality therapies for treatment of malignant pleural mesothelioma have been clinically explored in the past decades. In this regard, hyperthermic intrathoracic or intrapleural chemotherapy has been used as one of the multimodality therapies. The question addressed was In patients with malignant pleural mesothelioma who undergo macroscopic complete resection (MCR) does performing a Hyperthermic intrathoracic chemotherapy (HITOCH) lead to improvement in survival? The trial was registered in PROSPERO https://www.crd.york.ac.uk/prospero/ under registration number: CRD42024588823.

Three hundred five papers were found using the reported search, 21 were relevant to our topic and only 7 represented the best evidence to answer the clinical question. The authors, journal, date and country of publication, patient group studied, study type, relevant outcomes and results of these papers are tabulated. Six studies demonstrated a survival benefit for patients receiving HITOCH with a median survival ranging from 13–35 months in comparison to 11 -22.8 months for the non HITOCH group. Three out of the five studies compared extra-pleural pneumonectomy (EPP) to extended pleurectomy decortication (EPD) as the surgical preference for MCR. Only one study by Van Sandick et al. found a negative outcome with HITOCH in patients performing EPP (11 months vs 29 months). There was no reported mortality in relation to complications associated with HITOCH. The most common complication was atrial fibrillation followed by renal impairment. Despite the heterogeneity, small number of cases and lack of prospective randomised controlled trials, the body of evidence identified in this work demonstrates that HITHOC added to MCR in patients with pleural mesothelioma is safe and feasible. Possible improvement in recurrence free survival and overall survival warrant investigation in a randomised controlled trial.

**Supplementary Information:**

The online version contains supplementary material available at 10.1186/s12957-025-03748-8.

## Introduction

Surgical treatment of mesothelioma began to rise in the 1940s with the use of pneumonectomy and pleurectomy. Later in the 1960s, the pleurectomy and decortication procedure was introduced, a surgery that has been resurrected in the last decade. It was originally used back then on patients with trapped lung caused by infections mainly tuberculous empyema. In the 1970s, doctors experimented with another surgery called an extra-pleural pneumonectomy, which was also originally used to treat tuberculous empyema. The mortality rate for the surgery back then was as high as 31% [1] Today a thoracic surgeon can be involved in a wide variety of surgeries for mesothelioma ranging from a palliative intent reaching to the most aggressive form with an aim of macroscopic complete clearance.

Surgery-based multimodality therapies have been clinically explored in the past decades. In this regard, hyperthermic intrathoracic or intrapleural chemotherapy has been used as one of the multimodality therapies. Intrapleural injection of cytotoxic drugs with hyperthermic perfusion has been proved to enhance cytotoxic effect on tumor cells with limited systemic side effect. Potential mechanisms of hyperthermic intra-pleural or intraperitoneal chemotherapy are not only the tumor cells are directly exposed to higher concentration of chemotherapeutic agents, but also up to 44 °C for 1 h hyperthermic exposure render the cancer cells become more sensitive to the chemotherapeutic drugs while the normal tissues are unharmed [2]. In this study, we aim to compare macroscopic complete resection (MCR) procedures for malignant pleural mesothelioma (MPM) in the form of pleurectomy decortication (P/D)/extrapleural pneumonectomy EPP alone vs combined MCR procedures with hyperthermic intrathoracic chemotherapy (HITOCH) in terms of survival and complications.

### Search strategy

#### Study design and setting

This research constitutes a systematic review conducted in accordance with the guidelines of the JBI mixed-methods systematic reviews (MMSR) methodology, initially undertaken in June 2022. The authors tried to investigate the impacts of HITOCH on survival in MPM in patients undergoing MCR in comparison with patients undergoing MCR without HITOCH. This study adhered to the Preferred Reporting Items for Systematic Reviews and Meta-Analyses (PRISMA) to ensure the reliability and validity of the study results. The trial was registered in PROSPERO https://www.crd.york.ac.uk/prospero/ under registration number: CRD42024588823. The details are shown in supplement 1.

#### Data sourcing and study selection

In [patients with malignant pleural mesothelioma who undergo macroscopic complete resection] does [performing a Hyperthermic intrathoracic chemotherapy (HITOCH)] lead to [improved survival]?

Our methodology followed the reporting guidelines of Meta-analysis Of Observational Studies in Epidemiology (MOOSE) and Preferred Reporting Items for Systematic Reviews and Meta-Analyses (PRISMA) guidelines. We electronically ran a search on CENTRAL, MEDLINE/PubMed, Cochrane Library, and Scopus. On Pubmed, the word search used was from January 1990 to December 2023 using PubMed interface:

[(hyperthermic intrathoracic chemotherapy) or (intraoperative chemotherapy) or (intrapleural chemotherapy) and (pleural mesothelioma)] was performed.

Original articles written in English and with comparison between HITOCH and non HITOCH after surgery for mesothelioma only were included. We included studies with different types of radical surgery for mesothelioma if a comparison was available between HITOCH and non HITOCH cases.

Duplicate records were automatically removed from all uploaded retrieved citations in Covidence. The titles and abstracts were evaluated by two separate reviewers, who removed those that didn't fit the requirements for inclusion. Two reviewers read selected citations in full, with the grounds for exclusion being recorded in Covidence. Discussion or the participation of a third reviewer was used to settle disagreements among reviewers. A flowchart detailing the screening procedure was created in accordance with PRISMA-ScR recommendations for openness and reproducibility.

The exclusion criteria: duplicate studies, conference articles, articles with unavailable full texts or gray literature, articles not related to HITOCH, intervention studies or studies that did not have a design suitable for the objectives of this review. Although the term malignant pleural mesothelioma has been replaced by mesothelioma (which is exclusively malignant), we used the historical term in our search criteria.

#### Data extraction

Both quantitative and qualitative data were extracted from studies included in the review by 2 independent reviewers using a self-developed extraction tool. When necessary, the data extraction tools were modified to accommodate the differences of each included study, and modifications were detailed in the systematic review. The data extracted included specific details about the populations; study methods; theoretical framework, where applicable, survival and complications of relevance to the review question. Any disagreements that arose between the reviewers were resolved through discussion or with an additional third reviewer. Where necessary, authors of papers were contacted to request missing or additional data.

#### Quality assessment

Multiple critical appraisal tools were used because a variety of study designs were included. While qualitative studies were evaluated using the JBI critical appraisal tool for qualitative research, quantitative studies were evaluated using JBI critical appraisal tools for different study types. The methodological quality of research reports and pre-print publications were evaluated. The appraisal was conducted independently by two reviewers, with any disputes being settled by discussion or the participation of a third reviewer. Studies that didn't fulfill the minimum standards of quality were not considered. The results of Risk of Bias/Quality Assessment were placed into categories based on the sum of points given by each reviewer (Low Risk of Bias [LRB], Medium Risk of Bias [MRB], High Risk of Bias [HRB]).

### Search outcome

**Fig. 1:**
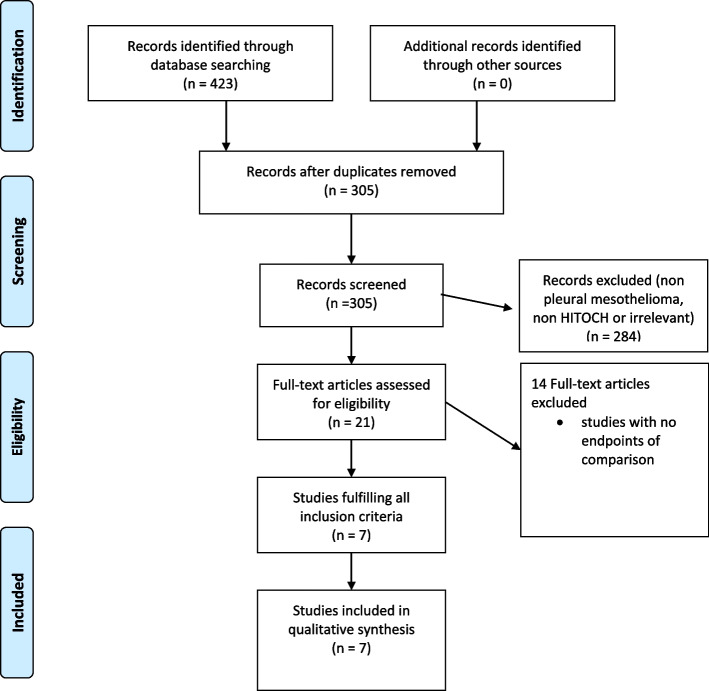
Flow diagram of study selection (PRISMA)

Three hundred and five papers were found using the reported search. Twenty-one papers were relevant to our topic and were assessed for full text. From these 7 papers were identified that provided the best evidence to answer the question. This is shown in PRISMA (Fig. 1). These are presented in Table 1.

**Table 1:** Best Evidence Papers

Author, date, journal and country, study type (level of evidence)	Patient group	Outcomes	Key results	Comments
[3] Van Sindick et al. (2008) Ann Surg Onc (Netherland) Retrospective study	20 patient who received HITOCH versus 15 patients who did not receive HITOCH = EPP/RT	Survival (HITOCH vs NO HITOCH) Chemotherapy agent used Complications and morbidity	(29 months for EPP/RT patients and 11 months for HITHOC patients) Intrathoracic temperature distribution of 40–41 °C. Cisplatin was given with a fixed dose of 80 mg/m2 and adriamycin with a dose starting at 20 mg/m2 Postoperative complications occurred in 8 EPP/RT patients (53%) and in 14 HITHOC patients (70%)	The only study to report an adverse outcome with HITOCH but is cofounded by different treatment modalities
[4] Tilleman et al (2009) J Thorac Cardiovasc Surg,	92 patients received HITOCH versus 29 patients who did not receive HITOCH	Survival (HITOCH vs NO HITOCH) Chemotherapy agent used Complications and morbidity	13.1 months vs 11 months Cisplatin (225 mg/m2) at 42 °C, with intravenous sodium thiosulfate for 60 min Morbidity (grade 3 or 4, 49%) included atrial fibrillation in 22 (23.9%) patients, venous thrombosis in12 (13%) patients, and laryngeal nerve dysfunction in 10 (11%) patients. 9 patients had renal toxicity, which was attributable to cisplatin in 8 of them. Among the 27 patients who also received amifostine 1 patient had grade 3 renal toxicity attributable to cisplatin	The largest study to compare HITOCH vs No HITOCH in patients with EPP
[5] Sugerbaker et al. (2013) J Thorac Cardiovasc Surg, USA	103 patients treated with cytoreductive surgery for MPM: 72 who received hyperthermic intraoperative cisplatin chemotherapy and 31 who did not	Survival (HITOCH vs NO HITOCH) Chemotherapy agent used Complications and morbidity	longer interval to recurrence (27.1 vs 12.8 months) and overall survival (35.3 vs 22.8 months) Cisplatin 175 to 225 mg/m2 for a 1 h lavage at 42 °C, with sodium thiosulfate rescue Of the 72 patients in the HITOC group, 27 (38%) were censored for recurrence. Of these 27 patients, 13 (18%) had died of other causes Of the 31 comparison patients, 23 (74%) developed recurrence, and 8 (26%) were censored: 4 (13%) were lost to follow-up, and 4 (13%) died of other causes	The improved interval to recurrence and overall survival for the hyperthermic intraoperative cisplatin chemotherapy group were particularly evident among the subgroups of patients who had not received hemithoracic radiotherapy and who had pathologic stage N1 or N2 lymph node metastases
[6] Isik et al (2013) Resp Med	9 patients with metastatic MPEs were treated with HIPEC following surgical interventions such as pleurectomy/decortication and/or lung resection comparison was done with historical control groups consisted of patients who received either talc pleurodesis or pleurectomy/decortication followed by systemic treatment	Survival (HITOCH vs NO HITOCH) Chemotherapy agent used Complications and morbidity	Median survival in group 1, 2 and 3 were 15.4, 6, 8 months, respectively. One year survival was 54.7% in group 1 where it was 0.6% and 0.8% in group 2 and 3, respectively 300 mg/m^2^ of IP cisplatin to all subjects There was no operative mortality. Morbidity was occurred in 1 patient in group 1 (5.3%)	This study included patients with malignant pleural effusion apart from MPM This study shows that IP HIPEC in combination with cytoreduction provides significantly better survival compared to those who received either pleurectomy or talc pleurodesis for the management of metastatic pleural malignancies
[7] Ishibashi et al. (2015) Gen Thorac Cardiovasc Surg	14 patients with MPM, 4 patients recieved P/D and intraoperative intrapleural hyperthermic cisplatin perfusion, followed by systemic chemotherapy 10 patients recieved trimodality treatment of EPP, systemic chemotherapy, and intensity modulated radiation therapy for hemithorax	Survival (HITOCH vs NO HITOCH) Chemotherapy agent used Complications and morbidity	There was no operative mortality in P/D group + HITOCH group or EPP group Postoperative complications occurred in 4 patients in P/D + HITOCH group, while they occurred in 7 patients in EPP group	The authors concluded that P/D and intraoperative intrapleural cisplatin perfusion achieved a favourable macroscopic resection in patients with MPM who were intolerable to EPP
[8] patel et al. (2019) Indian J Surg Oncol. 2019	5 patients who underwent MCR for MPM (4 EPP and 1 EPD) followed by HITOCH with cisplatin compared to control group patients undergoing EPP without HITOCH (3 patients)	Survival (HITOCH vs NO HITOCH) Chemotherapy agent used Complications and morbidity	9 months, four patients of the HITOCH group were alive, One patient in the non-HITHOC group was alive and disease-free at 24 months cisplatin at a dose of 100–150 mg/m2 Grade 3–4 complications were seen in one patient in the HITHOC group and none in the non-HITHOC group	HITOCH can be performed without increasing the morbidity of P/D or EPP. Most of these patients require multimodality treatment and are best managed by multidisciplinary teams
[9] Elsayed et al (2024) Updates in Surg Updates in Surg Egypt, Prospective study	55 patients. Thirty patients performed only EPD/PD while 25 consecutive patients had EPD/PD followed by HITHOC	Survival (HITOCH vs NO HITOCH)	The overall survival time in the HITOC group was 28 months (95% CI 21.5–34.5) vs 22 months (95% CI 17.5–26.5) in the surgery only group	The only study comparing EPD with and without HITOCH with no cofounding factors
		Chemotherapy agent used	Cisplatin (125 mg/m2) infused for 70 min at a temp of 40c-43c	
		Complications and morbidity	The 30-day mortality in the HITOC group was 0% vs 1 case (3.3%) in the surgery group. HITOC related transient complications occurred in 4/25 (16%) of the HITOC group	

## Results

Van Sandick et al. [3] from Netherlands reported that median survival time of overall survival and disease-free survival was longer in the patients treated with extra-pleural pneumonectomy (EPP) and postoperative hemi-thoracic radiation (RT) compared to the patients treated with EPP and intraoperative HITHOC (29 months for EPP/RT patients and 11 months for HITOCH patients). The findings of this study are cofounded by using different treatment modalities, but it is the only study to report a negative outcome with HITOCH.

Tilleman et al. [4] reported a phase II prospective study. They reported that total 96 of 121 (79%) enrolled patients underwent EPP, of whom 92 (76%) received hyperthermic intraoperative intrapleural cisplatin perfusion after EPP. The median overall survival of the 121 enrolled patients was 12.8 months, median survival of the 92 patients treated with HITOCH was 13.1 months, which was significantly longer than that of the 29 patients without hyperthermic intrapleural cisplatin perfusion (13.1 vs 11.0 months, P = 0.01). The authors concluded that adding HITOCH following EPP added a survival benefit.

Sugerbaker et al. [5] studied a cohort of 103 patients who had cytoreductive surgery for MPM between 2001 and 2009: 72 who received hyperthermic intraoperative cisplatin chemotherapy and 31 who did not. The groups were balanced for prognostic factors, except for the use of neoadjuvant chemotherapy (more common in the non HITOCH group). The hyperthermic intraoperative cisplatin chemotherapy group exhibited a significantly longer interval to recurrence (27.1 vs 12.8 months) and overall survival (35.3 vs 22.8 months) than the comparison group. The improved interval to recurrence and overall survival for the hyperthermic intraoperative cisplatin chemotherapy group were particularly evident among the subgroups of patients who had not received hemi-thoracic radiotherapy and who had no pathologic stage N1 or N2 lymph node metastases.

Isik et al. [6] compared 19 patients with metastatic MPEs who were treated with HIPEC following surgical interventions such as pleurectomy/decortication and/or lung resection (Group 1). Comparison was done with historical control groups which consisted of patients who received either talc pleurodesis or pleurectomy/decortication followed by systemic treatment for the management of metastatic MPEs (group 2 and 3).

This study shows that IP HIPEC in combination with cytoreduction provides significantly better survival compared to those who received either pleurectomy or talc pleurodesis for the management of metastatic pleural malignancies.

Ishibashi et al. [7] studied 14 patients with MPM were intended to treat with multimodality therapy including surgery. Four patients who were intolerable to EPP received a protocol consisting of P/D and intraoperative intrapleural hyperthermic cisplatin perfusion, followed by systemic chemotherapy. Ten patients received trimodality treatment of EPP, systemic chemotherapy, and intensity modulated radiation therapy for hemithorax. There was no operative mortality in P/D group + HITOCH group or EPP group. Postoperative complications occurred in 4 patients in P/D + HITOCH group, while they occurred in 7 patients in EPP group. Two-year DFS in P/D group was 75%, and median DFS did not reach. Two-year DFS in EPP group was 27%, and median DFS was 12.1 months. The authors concluded that P/D and intraoperative intrapleural cisplatin perfusion achieved a favorable macroscopic resection in patients with MPM in comparison to EPP.

Patel et al. [8] studied 5 patients who underwent MCR for MPM (4 EPP and 1 EPD) followed by HITOCH with cisplatin at a dose of 100–150 mg/m2. They compared this cohort to their contemporary control group patients undergoing EPP without HITOCH (3 patients).

Grade 3–4 complications were seen in one patient in the HITHOC group and none in the non-HITHOC group. At a median follow-up of 9 months, four patients of the HITOCH group were alive, three without recurrence, and one with recurrence. One patient in the non-HITHOC group was alive and disease-free at 24 months, and two died of progression at 18 and 36 months.

Elsayed et al. [9] studied 55 patients with localized pleural mesothelioma who underwent pleurectomy and decortication. Thirty patients performed only surgery while 25 consecutive patients had surgery followed by HITOCH with cisplatin (125 mg/m2) infused for 70 min at a temp of 40c-43c. The 30-day mortality in the HITOC group was 0% vs 1 case (3.3%) in the surgery group. HITOCH related transient complications occurred in 4/25 (16%) of the HITOCH group (atrial fibrillation, renal impairment and transient hypotension). Progression free survival in the HITOC group was 8 months (95% CI 4.3–11.6) vs 6 months (95% CI 2.5–9.9) in the surgery only group. The overall survival time in the HITOCH group was 28 months (95% CI 21.5–34.5) vs 22 months (95% CI 17.5–26.5) in the surgery only group. Risk factors analysis for recurrence in the HITOCH group confirmed a significant role for early stages (p = 0.03).

## Discussion

The advantages for using HITOCH after MCR for MPM are appealing and seem to attract more surgeons in recent years to use this facility. The idea of multimodality therapy (surgery + chemotherapy + hyperthermia) in one setting fits the idea of the general need of using multi-modality in patients with mesothelioma. The low systematic absorption and hence lower morbidity and more rapid recovery are additional advantages. The procedure is usually tolerated by most patients and is compatible with all adjuvant therapies.

The results of this study are interestingly presented in an era questioning the benefits of surgery beforehand for mesothelioma after the results of the MARS 2 trial have been recently released by Lim et al. [10]. OS favored chemotherapy alone for the first 42 months from randomization (hazard ratio [HR], 1.28; 95% CI, 1.02–1.60; P = 0.03). After 42 months, the difference in OS disappeared (HR, 0.48; 95% CI, 0.18–1.29; P = 0.15), Progression-free survival was not significantly different between the treatment arms (HR, 0.90; 95% CI, 0.72–1.11; P = 0.33). The risk of grade 3 or higher adverse events was greater for patients in the surgery arm than for those in the chemotherapy-alone arm (incidence rate ratio 3.6; 95% CI, 2.3–5.5; P < 0.001). Patients in the surgery arm had a greater risk of repeat interventions; cardiac disorders; infections or infestations; and respiratory, thoracic, or mediastinal disorders. Obviously, these results are a great discouragement to offer patients radical surgery for mesothelioma, but surgeons may still see benefits to offer a selected group of patients radical surgery for MPM.

Criticism of the trial includes enrolling patients with non-epithelioid pathology with known worse prognosis and results showing that 40% of patients in the surgery arm did not receive chemotherapy confounding the results to a comparison between surgery and chemotherapy for MPM. Also noted that there were key differences between the treatment arms at randomization when we look at the rate of diaphragmatic infiltration between the groups. Finally, nearly half (45%) of patients enrolled in MARS 2 trial were treated at centers with low surgical volumes. Patients in the surgical arm were deprived from the potential additional benefit of HITOCH.

The only study in our review that reports a negative outcome with HITOCH is the study by Van Sandick et al. [3]. The reasons for this were suggested by Miligore et al. [11] speculating that EPP is an aggressive surgery and the minimal invasiveness offered by VATS and P/D decreases perioperative complications and could serve to stimulate the immune system. The only two studies comparing the addition of HITOCH to the same procedure surgical cytoreductive procedure (EPD) with no cofounding of any other treatment factor were reported by Tilleman [4] with EPP and showed a survival benefit in favour of the HITOCH group (13.1 months vs 11 months), while EPD was reported by Elsayed et al. [9] and showed a median survival benefit in favour of the HITOCH group (28 months vs 22 months). All other studies [5–8] were cofounded by comparison of different cytoreductive procedures with or without HITOCH.

HITOCH may be considered as a safe, feasible and effective local treatment to improve the local effect of surgery, but even if many studies show promising results HITOCH has not been discussed in the last guidelines of the task force of the ERS/EACTS/ESTS/ESTRO on treatment of MPM, as Migliore and colleagues have already noticed [12].

The only attempt of randomization of HITOCH as a treatment modality was performed by Migliore and colleagues [13] as a pilot study and showed a survival benefit with VATS pleurectomy and HITOCH [13]. The criticism of the study lies in that the non HITOCH group performed only VATS talc pleurodesis, hence there is cofounding as one of the arms lack a cytoreductive procedure.

Designing a prospective randomized trial on large series of malignant pleural mesothelioma patients represents a major challenge itself because of the rarity of the tumor and its high mortality rate. Nevertheless, to face the imminent MPM incidence peak, now more than ever it’s urgent to create a standardized protocol including HITOCH, by joining all our efforts in more exhaustive, large and randomized studies. In fact, the needed samples size for a future trial, based on alfa significance level of 0.05 and 80% of power should be of 145 patients with approximately 73 events/deaths.

The project has obvious limitations, including those which are typical of any systematic review. By pooling observational studies, this review cannot overcome the limitations of its primary studies included which were relatively of small numbers and, still none were based on a randomized allocation. Indeed, the authors believe only meta-analyses of homogeneous well-powered randomized trials should be considered a solid scientific proof of the safety and efficacy of any medical/surgical intervention which is difficult to achieve on the short-term period in a rare disease like pleural mesothelioma and literature guidance from the available data is needed to support decision on a stretched medical resource setting in most countries. However, systematic reviews of non-randomized studies (as in the current case) can be meaningful and guide current practice, even if only by emphasizing the limitations of the available clinical evidence.

## Conclusion

Despite the heterogeneity, small number of cases and lack of prospective randomised controlled trials, the body of evidence identified in this work demonstrates that HITHOC added to MCR in patients with pleural mesothelioma is safe and feasible. Possible improvement in recurrence free survival and overall survival warrant investigation in a randomised controlled trial.

## Supplementary Information

Supplementary Material 1.

## Data Availability

No datasets were generated or analysed during the current study..
